# Accumulation mechanism of biofilm under different water shear forces along the networked pipelines in a drip irrigation system

**DOI:** 10.1038/s41598-020-63898-5

**Published:** 2020-04-24

**Authors:** Tianzhi Wang, Zucheng Guo, Yaojie Shen, Zhimei Cui, Alex Goodwin

**Affiliations:** 1 Environmental Simulation and Pollution Control State Key Joint Laboratory, School of Environment, Tsinghua University, Beijing, 100084 PR China; 2 China Everbright International LTD, China Everbright, Hongkong, 999077 China; 3 College of Water Resources and Civil Engineering, China Agricultural University, Beijing, 100083 China; 4 Department of Agricultural and Biological Engineering, University of Illinois at Urbana-Champaign, Urbana, IL 61801 USA

**Keywords:** Biofilms, Water microbiology

## Abstract

The behavior of clogging has a close relationship with the biofilm attached on inner surface of the pipeline in a drip irrigation system using reclaimed water. Therefore, inhibiting biofilm growth is the key to completely addressing the clogging problem. Water shear forces play a vital role in the formation, development and detachment of biofilm. In order to find out the accumulation mechanism of biofilm under different water shear forces, this paper considered 8 different shear forces with a range of [0, 0.7]Pa on the inner surface of pipelines in drip irrigation systems using three kinds of reclaimed water. The results indicate that dry weight (DW), phospholipid fatty acids (PLFAs) and extracellular polymeric substance (EPS) of biofilms show a S-type trend, the maximum contents were observed when τ was 0.2 Pa or 0. 35 Pa. Besides, the influence of water shear forces on biofilms is dual. The formation of biofilm is a dynamic stabilization process. When there is a relatively large shear force, it is favorable to the transport and renewal of microorganisms and nutrients. Meantime, the renewal speed of biofilms is also relatively fast. It is easy to form the biofilms with large surface and small thickness due to relatively high possibility of detachment. When the shear force is small, the transport speed of microorganisms and nutrients are limited, and the ability of microorganisms to secrete polysaccharides is reduced, which makes the nutrients needed for microbial growth insufficient and the adhesion between particles is also reduced, resulting in loose, unstable and an easily removed biofilm structure. After a comprehensive consideration of the dual influence, the critical controlling threshold of internal water shear force was obtained as [0, 0.20] ∪ [0.35, +∞] Pa. In addition, the growth model established in this paper can well describe the growth kinetics of attached biofilms, and provide theoretical reference for monitoring the occurrence of bio-clogging process in drip irrigation systems.

## Introduction

Drip irrigation is considered to be the safest and most reliable irrigation method for reclaimed water because of its precision and controllability. However, the reclaimed water still contains a large amount of particulate matter, microorganisms, organic matter, nitrogen and phosphorus, which greatly increases the risk of clogging in drip irrigation systems^1^. A large number of studies have found that when using reclaimed water, the clogging of drip irrigation systems has a close relationship with the formation and growth of biofilms attached on inner walls of the pipeline^2–5^. A common form of clogging in drip irrigation systems is that after being formed in the pipeline system, especially on inner walls of drip irrigation pipelines, the biofilms fall into the drip irrigation emitter and this results in clogging. Multiple studies have shown that the clogging is a difficult problem to solve^3–7^. At present, the problem of clogging in drip irrigation systems can be solved to some extent by properly setting up filtration equipment and system flushing^6–8^. The flushing of pipelines in drip irrigation system mainly promotes the rapid detachment of the attached biofilm by increasing the water shear forces of water flow in pipelines, which is an important way to control the clogging of drip irrigation systems.

Studies show that the formation of biofilms begins with microbial adhesion, in which microorganisms reach the substrate through physical movement, and the forces involved during this process mainly include diffusion, gravity, momentum and water shear forces^9^. In addition, mature biofilms are the result of simultaneous physical, chemical, and biological processes^10^. Based on the above issues, the hydrodynamic conditions have one of the primary roles in biofilm development and in determining biofilm stability and cohesion^11–14^. Firstly, the flow rate of water significantly affects the material transport mechanism^15^. Mathieu *et al*. found biofilms may detach and move with the flow or they may reattach or deposit in a downstream section^16^. Secondly, hydrodynamics also produce water shear forces that have a direct effect on deformation and detachment of biofilms. Van Loosdrecht *et al*. showed that the phenomenon that biofilm structures depend on the balance between substrate load and shear force is a balance between water shear forces and biofilm growth^17,18^. Other studies reported the correlation between the detachment rate of biofilms and the water shear force, which was found to be either exponential^11^, linear^19^, positively related to turbulence^20,21^ and shear forces^22,23^. This phenomenon leads to the water shear forces control threshold value on biofilm growth process. However, studies on the influence of water shear forces on biofilms mainly focused on biofilm reactors in municipal pipelines and the field of water treatment^9,18,24–26^. Studies on the influence of water shear forces on biofilms in drip irrigation systems have not been reported.

At present, the method of sampling attached biofilms in pipeline systems is mainly *in-situ* sampling. And some scholars have also proposed a variety of sampling methods, but these sampling methods are mainly used for large water supply pipelines (with a diameter usually more than 300 mm)^4–7^. For irrigation systems, the diameter of pipelines is usually small(typically between 12 mm and 25 mm)^5^. With a smaller diameter, the clogging of the irrigation systems has a larger impact, so the existing sampling device is obviously not applicable. Besides, if the *in-situ* destructive sampling not only reduce the number of attached biofilms, it also makes it difficult to reflect how various factors influence the accumulation mechanism of biofilms. It is very difficult to quantitatively study the growth of biofilms under specific shear forces because *in-situ* sampling only shows the result of multiple factors.

To address the challenges listed above, this paper has developed a culture device and sampling methods for the biofilms based on the hydrodynamic characteristics of networked pipelines in a drip irrigation system. In order to find out the accumulation mechanism of biofilm under different water shear forces, the paper quantitatively study the influence of water shear force on the inorganic component, organic component and microbial content of biofilms. And the paper considered 8 different shear forces with a range of [0, 0.7]Pa on the inner surface of pipelines in drip irrigation systems using three kinds of reclaimed water. In addition, the results of this study could provide theoretical reference for relieving clogging in drip irrigation systems.

## Materials and Methods

### Experimental materials

#### Source of water

This experiment uses three kinds of reclaimed water qualified upon treatment by rapid biochemical treatment technology (RBTT), sequencing batch aeration wastewater recycling (SBWL) and cyclic activated sludge system (CASS) as sources of water. The water sources to be tested are stored in water storage tanks. Before the test system runs, the experimental water source in the water storage tank is evenly stirred and then replenished into water supply tank of the device to supplement the loss due to evaporation and splashing. 500 mL of different water samples stored at a constant temperature of 4 °C are taken and measured for water quality at PONY Testing International Group Beijing laboratory, with test results shown in Table 1.

**Table 1:** Water Quality Results (mg/L).

Water-quality Index (mg/L)	CASS	RBTT	SBWL
TSS	18.7	22.8	25.3
TOC	6.96	3.12	5.38
TN	22.7	23.2	28.5
TP	3.96	2.81	1.43
COD_cr_	25.5	8.3	18.2
BOD_5_	10.99	3.60	9.48
CO_3_^2−^ + HCO_3_^−^	337.4	213.2	232.4
PO_4_^3−^	3.45	2.43	1.36
Ca^2+^	37.6	12.1	67.5
Mn^2+^	<0.001	<0.001	0.028
Mg^2+^	13.4	8.87	26.0
Fe^3+^	0.25	0.28	0.98
Cl^−^	100	50.3	106
SO_4_^2−^	85.5	90.0	91.1
TPC (CFU/mL)	3.8 × 10^4^	8.2 × 10^4^	9.3 × 10^4^

#### Simulation system

**Figure 1:**
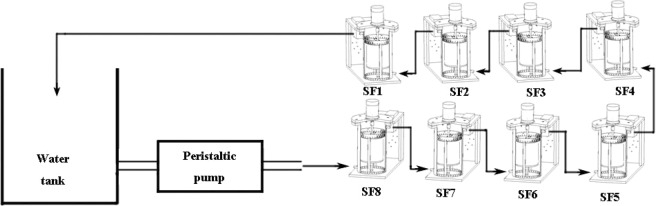
Test System and Simulation System.

**Figure 2:**
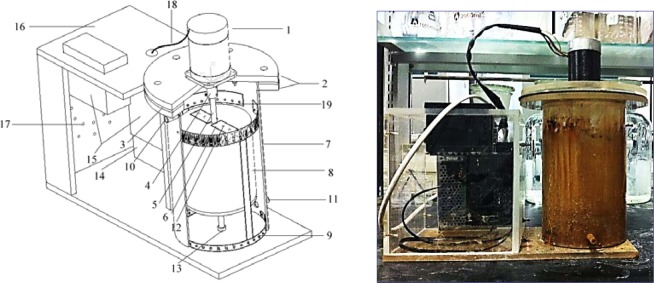
Simulator. Note: 1—Motor; 2—Flange plate; 3—Sheet gasket; 4—Connecting bearing; 5—Motor shaft; 6—Steel sheet; 7—Outer cylinder; 8—Inner cylinder; 9—Sampling frame; 10—Outlet; 11—Inlet; 12—Screw; 13—Fixing bearing; 14—Transformer; 15—Speed control device; 16—Distribution box; 17—Vents; 18—Wire; 19—Sample tank.

This experiment consists of reclaimed water simulation systems under three kinds of processing conditions. Each simulation system is composed of a parallel connection of water tanks, peristaltic pumps, latex tubes, drip irrigation system simulators. The water is stored in the water tank to provide water for the system. The peristaltic pump provides working driving force for the system (BT100L; Baoding, China), and is connected via latex tube to the water tank. The experiment sticks onto a PE pipeline section onto the inner wall of the outer cylinder (7) of the reactor. In order to simulate the wall medium of a real drip irrigation system, the pipeline section is taken from commonly used Φ16 drip irrigation pipeline with a size of 19 cm × 1 cm. When the simulator motor is working, the rotation of the inner cylinder drives the water to flow, which will drive the water on the inner wall of the outer cylinder to flow. Since the sampling frame (9) with the PE section is fixed on the inner wall of the outer cylinder(7), shear forces will occur on the surface of the PE section. The simulators (SF1 to SF8) are connected in series, and the peristaltic pump is connected to the inlet (11) of SF8 simulator through a latex tube. The outlet of SF8 simulator is connected to the inlet of SF7 simulator through a latex tube (10)…outlet (11) of SF2 simulator is connected to the outlet (10) of SF1 simulator through a latex tube, and the peristaltic pump pumps water to the inner and outer cylinders of all simulators. After they are filled, the water flows from outlet of SF1 simulator through the latex tub to the water tank. The series connection is shown in Fig. 1, and the simulators and their parameters are shown in Fig. 2 and Table 2, respectively. The background and introduction of the simulation system designed can be found in Appendices.

**Table 2:** Simulator Parameters.

Outer Cylinder Size (mm)	Inner Cylinder Size (mm)	Inlet Size (mm)	Outlet Size (mm)	Motor Power	Rotational Speed
Φ130*252	Φ110*135	ID Φ10	ID Φ10	150w	0–3000r/min

#### Simulation of water shear forces at different positions of drip irrigation pipeline

**Figure 3:**
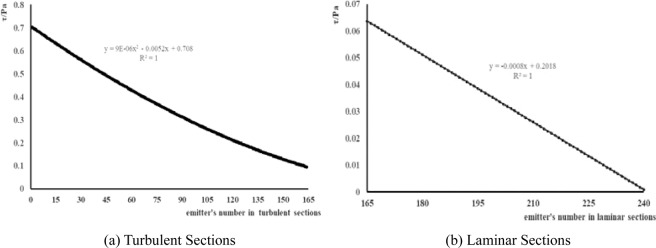
Characteristics of Changes in Water Shear Force along Pipelines within Drip Irrigation Belt.

The calculation of actual pipeline shear force is determined according to the calculation method of pipeline hydraulics in fluid mechanics^27^ and the design of drip irrigation^28^. This test simulates the hydraulic conditions within the pipeline when the field drip irrigation tube is 80 m long. The flow rate of simulated emitters is 1.2 L/h, and the distance between emitters is 0.3 m. A total of 240 emitters are divided into two sections as shear forces change along the length of the pipeline: The 1st through the 164th sections are turbulent sections, while the 165th through the last sections (the 240th section) are laminar sections, with characteristics of changes in water shear force at each section shown in Fig. 3.

#### Calculation and control of water shear forces of simulators

Simulators used in this test recommend a hydraulic retention time of 2 h (Peter *et al*., 2003) by lower inlet and upper outlet. When the total volume of simulators is 1210 mL, the water inflow is 10.08 mL/min, and the flow rate is very low and could be ignored. And the calculation of shear forces of water flow on the wall surface in the reactor can be calculated by the calculation of flow between the coaxial rotating cylinders in Viscous Fluid Mechanics^27^, as Eq. 1:

1 $${\tau }_{r}=-\,2\mu \times \frac{{R}_{1}^{2}\cdot {R}_{2}^{2}}{({R}_{2}^{2}-{R}_{1}^{2})}\times \frac{({\omega }_{1}-{\omega }_{2})}{{r}_{2}^{2}}$$

Where: *τ*_*r*_ represents the frictional stress (N/m^2^) at the radius *r*, the direction of which is opposite to the angular velocity ω_1_; R_1_ and R_2_ are the radius (m) of inner and outer cylinders, respectively; ω_1_ and ω_2_ are the angular velocity (rad /s) of inner and outer cyllinders respectively; r_2_ represents the radius of a circular section between the inner and outer cylinders; μ is the liquid viscosity coefficient (N•s/m^2^), which is related to the type of liquid and its temperature.

Combined with the specific conditions in this test, ω_2_ = 0 rad/s; r_2_ = R_2_; since the frictional stress is the frictional force of the wall against the water flow, and the water shear force of the water flow to the wall surface is discussed here, Eq. 1 can be simplified to:

2 $$\tau =2\mu \times \frac{{{R}_{1}}^{2}\cdot {\omega }_{1}}{({{R}_{2}}^{2}-{{R}_{1}}^{2})}$$

i.e.:

3 $${\omega }_{1}=\frac{\tau \times ({{R}_{2}}^{2}-{{R}_{1}}^{2})}{2\mu \cdot {{R}_{1}}^{2}}$$

Each symbol in Eq. (2) and Eq. (3) is identical to Eq. 1, and μ is the dynamic viscosity coefficient of water at 20 °C, which is 1.005 × 10^−3^ (N•s/m ^2^).

Formula 3 is the calculation formula for water shear forces on the inner wall surface of the outer cylinder of the test simulator.

It can be seen from Table 2 that R_1_ = 65 mm; R_2_ = 55 mm; μ = 1.005 × 10^−3^(N•s/m^2^); therefore, the formula for calculating the water shear force on inner wall of the outer cylinder of the simulator is:

4 $${\omega }_{1}=\frac{\tau \times 1200}{6080.25}\times {10}^{3}$$

In the experimental shear force setting, loci of 6 turbulent sections and 2 laminar sections are selected. The shear forces and rotational speed of simulators for this test are set according to the water shear force simulation results at different drip irrigation pipeline positions and the water shear force calculation results of simulators, as shown in Table 3.

**Table 3:** Simulator Shear Force and Rotational Speed.

	SF1	SF2	SF3	SF4	SF5	SF6	SF7	SF8
	Turbulent Sections		Laminar Sections					
**Reynolds number**	1418	1996	3072	3493	3887	4228	5200	6303
**Flow rate in pipe(m/s)**	0.090	0.126	0.194	0.220	0.245	0.267	0.328	0.398
**Shear force (Pa)**	0.05	0.10	0.20	0.25	0.30	0.35	0.50	0.70
**Reactor rotational speed(r/min)**	84	168	335	420	506	587	843	1181
**Distance from head end of pipeline(m)**	56.1	49.5	37.2	32.4	27.9	24.0	12.9	0

### Sampling and testing methods of biofilms

During the experiment, the system operated 8 h everyday (8:00 am–12:00 pm, 3:00 pm–7:00 pm), and the experiment lasted for 100 days (800 h in total). The samples were collected every 10 days (10 times in total). Two polyethylene sections were collected each time to test the DW, PLFAs and EPS according to the testing methods used by Zhou *et al*.^5^.

### Modeling of growth kinetics

Since the main material clogging drip irrigation systems under reclaimed water conditions is the attached biofilm formed by microbial growth on the wall surface (Zhou 2016), the following assumptions are made: (1) net growth ${Y}_{1}$ of biofilm is assumed to be in accordance with a Logistic growth model^29,30^; (2) it is assumed that there is a positive correlation between the detachment amount and the amount of growth of the biofilm, and there is a linear or exponential relationship between these two; and (3) it is assumed that the net growth process of biofilm is linearly related to the shear force^19^.

According to the assumption (1), net growth ${Y}_{1}$ of biofilm can be obtained,

5 $${Y}_{1}=\frac{{y}_{max}}{1+{b}_{1}\times {e}^{-{b}_{2}\times T}}$$

According to the assumption (2), detachment amount ${Y}_{2}$ of biofilm can be obtained,

6 $${Y}_{2}={b}_{3}\times {\left(\frac{{y}_{max}}{1+{b}_{1}\times {e}^{-{b}_{2}\times T}}\right)}^{{b}_{4}}$$

According to the assumption (3), net growth ${Y}_{1}$ and detachment amount ${Y}_{2}$ of biofilm can be obtained, respectively,

7 $${Y}_{1}={b}_{5}\times \tau \times (\frac{{y}_{max}}{1+{b}_{1}\times {e}^{-{b}_{2}\times T}})$$

8 $${Y}_{2}={b}_{3}\times {\left({b}_{5}\times \tau \times (\frac{{y}_{max}}{1+{b}_{1}\times {e}^{-{b}_{2}\times T}})\right)}^{{b}_{4}}$$

Growth amount $Y$ of biofilm is the difference between net growth and detachment amount,

9 $$Y={Y}_{1}-{Y}_{2}={b}_{5}\times \tau \times (\frac{{y}_{max}}{1+{b}_{1}\times {e}^{-{b}_{2}\times T}})-{b}_{3}\times {\left({b}_{5}\times \tau \times (\frac{{y}_{max}}{1+{b}_{1}\times {e}^{-{b}_{2}\times T}})\right)}^{{b}_{4}}$$

Where: $Y$ is the growth amount of attached biofilm per unit area; ${Y}_{1}$ is the net growth of attached biofilm per unit area; ${Y}_{2}$ is the detachment amount of attached biofilm per unit area; τ is the water shear force (Pa); ${y}_{max}$ is the maximum capacity per unit area in the attached biofilm environment, that is, the maximum value of components; $T$ is the growth time of biofilm (h); ${b}_{1}$, ${b}_{2}$, ${b}_{3}$, ${b}_{4}$ and ${b}_{5}$ are model’s fitting parameters.

## Results and analyses

### Dynamic change characteristics of DW

**Figure 4:**
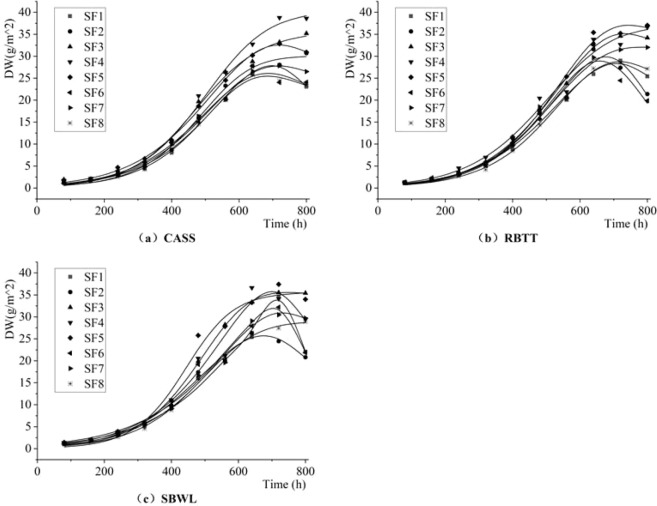
Dynamic Change Process of Dry Weight of the Attached Biofilm per Unit Area on the Inner Wall of the Drip Irrigation Pipeline.

Figure 4 shows the dynamic change characteristics of dry weight (DW) of attached biofilm per unit area on inner wall of drip irrigation pipeline under different shear forces. Table 4 shows the results of fitting parameters obtained by nonlinear fitting formula (9) of the relationship between dry weight and growth time of biofilm by using *1stopt software*. It can be seen from the table that determination coefficient R^2^ of the fitting function is above 0.97, indicating that the fitting effect is good, and that F-value of the F-test (α = 0.05) is greater than 334 (more than 4.8), indicating that the fit function has passed the test.

**Table 4:** Fitting Parameters of the Growth Dynamics Model for Dry Weight of Attached Biofilm per Unit Area on the Inner Wall of the Drip Irrigation Pipeline.

		b1	b2	b3	b4	b5	R2	F
CASS	0.05 Pa	366	0.0080	5.3 × 10^−2^	1.58	127	0.99	1552
	0.10 Pa	867	0.0114	9.7 × 10^−1^	1.00	2813	1.00	2728
	0.20 Pa	125	0.0098	1.9 × 10^−4^	2.76	6	1.00	2200
	0.25 Pa	148	0.0091	4.7 × 10^−4^	2.59	6	1.00	1930
	0.30 Pa	203	0.0078	3.3 × 10^−2^	1.66	11	1.00	5258
	0.35 Pa	676	0.0096	9.9 × 10^−1^	1.00	2742	0.99	873
	0.50 Pa	260	0.0084	5.4 × 10^−2^	1.57	8	1.00	2702
	0.70 Pa	1051	0.0106	7.1 × 10^−1^	1.05	56	1.00	3359
RBTT	0.05 Pa	326	0.0079	8.6 × 10^−2^	1.46	134	0.99	1357
	0.10 Pa	104	0.0082	2.0 × 10^−11^	7.40	15	0.99	662
	0.20 Pa	103	0.0084	1.2 × 10^−12^	7.87	7	1.00	4216
	0.25 Pa	69	0.0081	1.0 × 10^−9^	5.99	5	0.99	609
	0.30 Pa	137	0.0083	4.0 × 10^−7^	4.43	4	0.99	1688
	0.35 Pa	1557	0.0104	9.9 × 10^−1^	1.00	7251	0.97	334
	0.50 Pa	254	0.0085	6.5 × 10^−2^	1.51	7	0.99	1088
	0.70 Pa	269	0.0083	1.0 × 10^−2^	1.97	5	1.00	6161
SBWL	0.05 Pa	69	0.0067	3.1 × 10^−48^	30.49	30	0.99	1426
	0.10 Pa	74	0.0089	9.8 × 10^−24^	16.16	12	0.99	1263
	0.20 Pa	176	0.0094	4.1 × 10^−5^	3.27	8	1.00	11957
	0.25 Pa	99	0.0076	1.0 × 10^−9^	6.04	7	0.98	381
	0.30 Pa	294	0.0120	8.3 × 10^−5^	3.06	3	0.99	636
	0.35 Pa	2672	0.0076	6.5 × 10^−1^	1.06	476	0.99	598
	0.50 Pa	615	0.0087	4.3 × 10^−1^	1.13	30	0.99	1416
	0.70 Pa	188	0.0090	1.0 × 10^−2^	1.96	3	1.00	3080

Overall, the DW of biofilm shows an “S-type” growth trend with the cumulative operation of the system, which can be divided into three phases: adaptive phase, rapid growth phase and dynamically stable phase. In the first 240 h that the system works, the growth of attached biofilm is in the adaptive phase, and the overall growth of DW is slow. At the end of this phase, the average DW of attached biofilm is 3.43 g/m^2^. During the period of 240~640 h, the DW of biofilm increases rapidly. When the system works for 640 h accumulatively, the average DW of biofilm increases to 29.71 g/m^2^. After that, the DW of biofilm tends to be dynamically stable with a stable value of 28.68 g/m^2^ (800 h). For three different reclaimed water sources, the average DW difference of attached biofilm on inner walls of drip irrigation pipeline is (0.38 ± 2.76%) g/m^2^, respectively. When in the dynamically stable phase, the DW of biofilm under reclaimed water treated by RBTT is the largest (30.25 g/m^2^), which is 4.40% and 1.29% higher than that under reclaimed water treated by CASS and SBWL, respectively.

For different shear forces, during the adaptive phase, the DW of biofilm increases first and then decreases with the increase of shear force. In the range of [0.05, 0.25]Pa, the DW of biofilm increases with the increase of shear force. But in the range of [0.25, 0.70]Pa, the DW of biofilm decreases with the increase of shear force. At the end of this phase (240 h), the DW of biofilm under the 0.25 Pa is 3.99 g/m^2^, which is 1.15%~51.52% higher than that under other shear conditions. As the most important and fastest phase of biofilm growth, the growth rate of DW of biofilm also increases first and then decreases with the increase of shear force, and reaches the highest of 0.08 g/m^2^/h under 0.25 Pa of shear force, which is 14.29%~33.33% faster than that under other shearing conditions. During the dynamically stable phase, the DW of biofilm in shear force range of [0.20,0.35]Pa is the largest, with a mean value of 34.18 g/m^2^, which is 18.18% and 17.91% higher than that in shear force range of [0.05,0.20]Pa and [0.35,0.70]Pa, separately.

### Dynamic change characteristics of PLFAs

**Figure 5:**
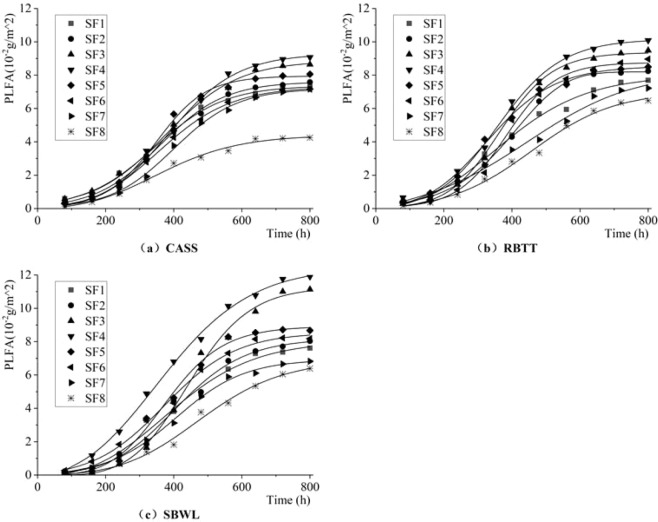
Dynamic Change Process of PLFAs in the Attached Biofilm per Unit Area on Inner Wall of Drip Irrigation Pipeline.

Figure 5 shows the dynamic change characteristics of phospholipid fatty acids (PLFAs) of the attached biofilm per unit area on the inner wall of the drip irrigation pipeline under different shear forces. Table 5 shows the results of the fitting parameters obtained by the nonlinear fitting formula (9) of the relationship between phospholipid fatty acids and the growth time of the biofilm by using *1stopt software*. It can be seen from the table that the determination coefficient R^2^ of the fitting function is above 0.99, indicating that the fitting effect is good, and that F-value of the F-test (α = 0.05) is greater than 907 (more than 4.8), indicating that the fit function has passed the test.

**Table 5:** Fitting Parameters of the Growth Dynamics Model for PLFAs in the Attached Biofilm per Unit Area on the Inner Wall of the Drip Irrigation Pipeline.

		b1	b2	b3	b4	b5	R2	F
CASS	0.05 Pa	29	0.0098	3.6 × 10^−19^	−29.42	20	1.00	4909
	0.10 Pa	45	0.0106	1.5 × 10^−3^	−2.61	10	1.00	4461
	0.20 Pa	34	0.0094	1.6 × 10^−16^	−25.33	5	1.00	3473
	0.25 Pa	35	0.0096	7.4 × 10^−1^	0.70	6	1.00	2586
	0.30 Pa	107	0.0126	1.7 × 10^−3^	3.03	3	1.00	3302
	0.35 Pa	18	0.0089	1.0 × 10°	1.00	319	1.00	18014
	0.50 Pa	63	0.0106	9.9 × 10^−1^	1.00	93	1.00	3889
	0.70 Pa	11	0.0078	1.0 × 10°	1.00	230	0.99	1692
RBTT	0.05 Pa	9	0.0071	1.0 × 10°	1.00	24566	0.99	1660
	0.10 Pa	327	0.0111	8.0 × 10^−1^	1.03	313	1.00	9163
	0.20 Pa	81	0.0125	1.0 × 10°	1.00	587	1.00	5256
	0.25 Pa	30	0.0102	1.0 × 10°	1.00	1761	1.00	2498
	0.30 Pa	18	0.0096	1.0 × 10°	1.00	270	1.00	3280
	0.35 Pa	220	0.0134	1.0 × 10^−2^	2.24	2	1.00	2155
	0.50 Pa	18	0.0064	2.1 × 10^−1^	−0.90	2	0.99	1341
	0.70 Pa	44	0.0082	9.5 × 10^−2^	−0.32	2	1.00	1843
SBWL	0.05 Pa	11	0.0073	1.0 × 10°	1.00	27354	0.99	907
	0.10 Pa	34	0.0094	1.0 × 10°	1.00	546	0.99	1188
	0.20 Pa	74	0.0104	1.0 × 10°	1.00	532	1.00	1948
	0.25 Pa	8	0.0068	1.1 × 10°	0.99	161	1.00	5342
	0.30 Pa	43	0.0110	1.0 × 10°	0.99	117	0.99	1608
	0.35 Pa	34	0.0094	2.21.0 × 10^−1^	0.01	2	1.00	2934
	0.50 Pa	59	0.0100	3.01.0 × 10^−2^	−0.67	2	1.00	4630
	0.70 Pa	27	0.0076	1.0 × 10°	1.00	260	0.99	1475

As shown in Fig. 5, there is a difference in the change process between PLFAs and DW. In the first 160 h that the system works, the growth of PLFAs in the attached biofilm is in the adaptive phase, with an average growth rate of only 3.75 × 10^−5^g/m^2^/h. After the rate increases to 0.60 × 10^−2^g/m^2^, the PLFAs in the biofilm increases rapidly, entering the rapid growth phase(160~640 h) with its average growth rate increased to 14.61 × 10^−5^g/m^2^/h. When the system works for 640 h cumulatively, the mean value of PLFAs increases to 7.61 × 10^−2^g/m^2^. Thereafter, the PLFAs in biofilm tend to be dynamically stable with a stable value of 8.11 × 10^−2^g/m^2^ (800 h). For three different reclaimed water sources, the average PLFAs of attached biofilm on inner walls of drip irrigation pipeline is (0.38 ± 8.74%) × 10^−2^g/m^2^, respectively. In the dynamically stable phase, the PLFAs in the biofilm under reclaimed water treated by SBWL is the largest (8.32 × 10^−2^g/m^2^), which is 14.74% and 2.30% higher than that under reclaimed water treated by CASS and RBTT, respectively.

For different shear forces, during the adaptive phase, the PLFAs content in biofilm is higher under 0.25 Pa shear force than that under other shear forces. At the end of this phase (160 h), the average value of PLFAs in biofilm under the 0.25 Pa shear force is 0.73 × 10^−2^g/m^2^, which is 7.08%~145.58% higher than that under other shear conditions. During the rapid growth phase, the content and growth rate of PLFAs in biofilm gradually show a trend in which increasing the shear force the content and growth rate of PLFAs increase first and then decrease in the range of [0.25,0.70]Pa the content and growth rate of PLFAs in biofilm decease with the increase of shear force, and under 0.25 Pa of shear force the growth rate reaches its peak of 18.55 × 10^−5^g/m^2^/h which is 5.32%~84.37% faster than that of PLFAs in biofilm under other shear forces. During the stable phase, the content of PLFAs in the biofilm in the range of [0.20,0.35]Pa shear force is the largest, with a mean value of 9.29 × 10^−2^g/m^2^, which is 13.38% and 30.08% higher than that in shear force range of [0.05,0.20]Pa and [0.35,0.70]Pa, respectively; and among them, the highest average value of PLFAs in the biofilm is 10.07 × 10^−2^g/m^2^ under 0.25 Pa shear force, which is 6.37%~84.59% higher that than under other shear forces.

### Dynamic change characteristics of EPS

**Figure 6:**
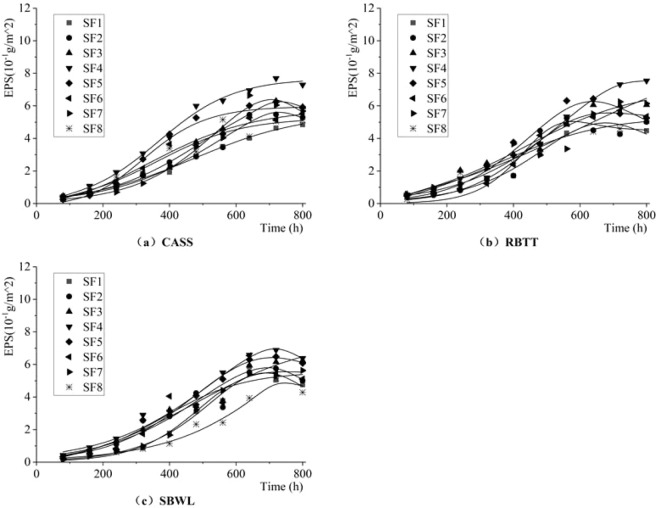
Dynamic Change Process of EPS in the Attached Biofilm per Unit Area on the Inner Wall of the Drip.

Figure 6 shows the dynamic change characteristics of extracellular polymeric substance (EPS) of the attached biofilm per unit area on inner wall of the drip irrigation pipeline. Table 6 shows the results (R^2^  > 0.91 and F > 96, with a significance level of a = 0.01) of fitting parameters obtained by the nonlinear fitting formula (9) of the relationship between extracellular polymeric substance and growth time of biofilm by using *1stopt software*.

**Table 6:** Fitting Parameters of the Growth Dynamics Model for EPS in the Attached Biofilm per Unit Area on the Inner Wall of Drip Irrigation Pipeline.

		b1	b2	b3	b4	b5	R2	F
CASS	0.05 Pa	18	0.0061	1.7 × 10^−2^	−2.35	24	0.99	1317
	0.10 Pa	33	0.0061	1.3 × 10^−1^°	12.17	15	0.98	452
	0.20 Pa	30	0.0067	1.5 × 10^−56^	67.08	6	0.99	1700
	0.25 Pa	26	0.0089	3.4 × 10^−2^	−1.70	4	0.99	1606
	0.30 Pa	18	0.0100	1.0 × 10°	1.00	560	0.97	287
	0.35 Pa	5	0.0060	1.0 × 10°	1.00	2173	0.99	651
	0.50 Pa	818	0.0087	1.0 × 10^0^	1.00	9147	0.98	448
	0.70 Pa	18	0.0079	4.1 × 10^−2^	−1.56	1	0.95	183
RBTT	0.05 Pa	58	0.0072	3.1 × 10^−4^	4.55	34	0.97	273
	0.10 Pa	2787	0.0150	7.7 × 10^−1^	1.05	321	0.96	242
	0.20 Pa	14	0.0060	1.0 × 10^−1^	−2.11	6	0.98	389
	0.25 Pa	36	0.0061	2.5 × 10^−7^	7.09	6	1.00	1885
	0.30 Pa	47	0.0085	1.0 × 10^−9^	10.49	3	0.97	275
	0.35 Pa	97	0.0086	1.0 × 10^−2^	2.66	4	0.99	997
	0.50 Pa	12	0.0042	3.9 × 10^−1^	−1.58	2	0.95	170
	0.70 Pa	11	0.0065	1.6 × 10^−3^	−1.32	2	0.96	208
SBWL	0.05 Pa	109	0.0080	9.0 × 10^−5^	5.01	34	0.98	477
	0.10 Pa	24	0.0083	6.8 × 10^−34^	0.67	10	0.94	150
	0.20 Pa	14	0.0054	4.6 × 10^−1^	−0.38	6	0.98	369
	0.25 Pa	25	0.0058	1.0 × 10^−9^	10.01	6	0.98	454
	0.30 Pa	38	0.0075	1.0 × 10^−9^	10.38	3	0.99	781
	0.35 Pa	193	0.0067	3.9 × 10^−1^	1.21	28	0.91	96
	0.50 Pa	190	0.0094	1.0 × 10^−2^	2.66	3	1.00	2825
	0.70 Pa	3000	0.0051	1.0 × 10^−2^	2.74	141	0.98	439

From the results of Fig. 6 and Table 6, the overall trend of EPS is still similar to that of DW and PLFAs, which can be divided into three phases successively. The EPS grows slowly in the first 160 h that the system works, with an average growth rate of 4.38 × 10^−4^g/m^2^/h. At the end of this phase, the average EPS value reaches 0.70 × 10^−1^g/m^2^; from 160~640 h, the EPS grows rapidly, with an average growth rate of 10.16 × 10^−4^g/m^2^/h. After that, the EPS tends to be dynamically stable, with a stable value of 5.58 × 10^−1^g/m^2^ (800 h). For three different reclaimed water sources, the average EPS in attached biofilm on inner walls of drip irrigation pipeline is (0.19 ± 6.47%) × 10^−1^g/m^2^, respectively. And in the dynamically stable phase, the EPS in the biofilm under reclaimed water treated by CASS is the largest (5.67 × 10^−1^g/m^2^), which is 2.42% and 0.14% higher than that under reclaimed water treated by RBTT and SBWL, respectively.

For different shear forces, during the adaptive phase, the EPS content in biofilm is higher under 0.25 Pa shear force than that under other shear forces. At the end of this phase (160 h), the average value of EPS in the biofilm under the 0.25 Pa shear force is 0.93 × 10^−1^g/m^2^, which is 24.45%~75.81% higher than that under other shear conditions. During the rapid growth phase, the content and growth rate of EPS in biofilm gradually show a trend in which they increase first and then decrease with the increase of shear force. Under 0.30 Pa of shear force, the growth rate of EPS reaches its peak of 11.92 × 10^−4^g/m^2^/h, which is 0.44%~67.64% faster than that under other shear forces. Until the dynamically stable phase, the content of EPS in the range of [0.20,0.35]Pa shear force is the largest, with a mean value of 6.34 × 10^−1^g/m^2^, which is 19.32% and 15.29% higher than that in shear force range of [0.05, 0.20]Pa and [0.35, 0.70]Pa, respectively. Among them, the highest mean value of EPS is 7.00 × 10^−1^g/m^2^ under 0.25 Pa shear force, which is 16.12%~50.66% higher that than under other shear forces.

## Discussion

**Figure 7:**
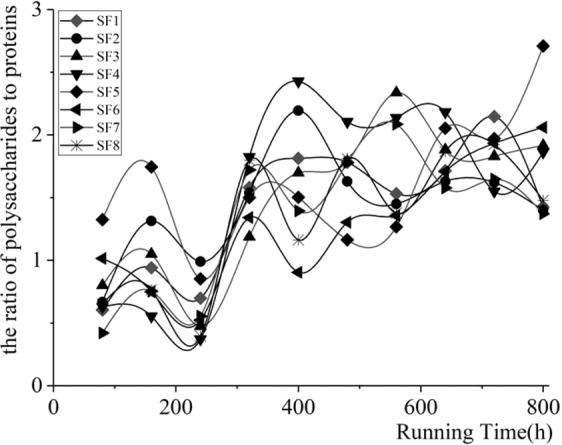
Ratio of Polysaccharides to Protein in Biofilm (All three kinds of water resource).

As the most important force of hydrodynamics in drip irrigation pipelines, water shearing force plays a vital role in the formation, development and of attached biofilms on inner walls in drip irrigation systems under reclaimed water. In order to find out the mechanism of how water shear forces influence the attached biofilm, this paper has found that the DW of biofilm increases first and then decreases with the increase of shear force, and in the range of [0.20, 0.35] Pa shear force, the DW of biofilm reaches its peak. This is consistent with other studies that “the condition that maximum biofilm adhesion appears in microchannel is within the range of shear forces in the middle^31^.” This is mainly because the material of PE cultivate piece is hydrophobic, which could facilitate the initial adhesion of microorganisms^32^. The water shear force can promote the adhesion of microorganisms on inner surface^9^, further increasing the number of microorganisms adhering to the wall at the initial stage of biofilm formation. In the adaptive phase, the PLFA content in [0.4, 0.7] Pa shear force is higher than that in [0, 0.25] Pa shear force, which confirms ‘the condition that maximum biofilm adhesion appears in microchannel is within the range of shear forces in the middle’^31^. Therefore, the EPS secreted by the microorganism is also a highly hydrophobic binder^33^, which not only further promotes the adhesion of microorganisms onto the inner wall of the runner but also facilitates the adhesion of suspended particles in the water onto the wall surface. At the same time, shear forces alter some of the biological metabolic processes of microorganisms^34^, and high shear forces significantly stimulate microbial respiration^34^, while low shear forces can cause some nutrient and oxygen migration problems, leading to the lack of nutrients and oxygen required by microorganisms^31^. In addition, the increase in shear forces promotes the decomposition of microorganisms and produces more energy that will not be used to grow but to secrete polysaccharides^34^, which in turn changes the ratio of polysaccharides to protein and makes the same ratio increase as the shear force increases. As shown in Fig. 7, the experimental results show that the ratio of polysaccharides to protein gradually increases under the action of shear force, which is consistent with the study of Tay *et al*.^35^. As shown in Fig. 7, the experimental results in this paper show that under the action of shear force, the ratio of polysaccharides to protein increases gradually, which is consistent with the previous analysis.

In addition, with the increase of shear force, the ratio of polysaccharide to protein increases first, and then decreases. In the range of [0, 0.35] Pa shear force, the ratio of polysaccharides to protein increases with the increase of shear force, while in the range of [0.35, 0.70] Pa shear force, the ratio of polysaccharides to protein shows a tendency to decrease with an increase of shear force. This is mainly because under conditions of high shear force, due to the reversible initial adhesion of microorganisms, some microorganisms adhering to the surface of the medium will detach, leaving only some of microorganisms more firmly adhered on the surface of the medium^31^. The contents of microbial PLFAs and EPS in the initial biofilms under the condition of 0.25 Pa shear force are higher than those under other shear forces, which shows the consistency with other results.

**Figure 8:**
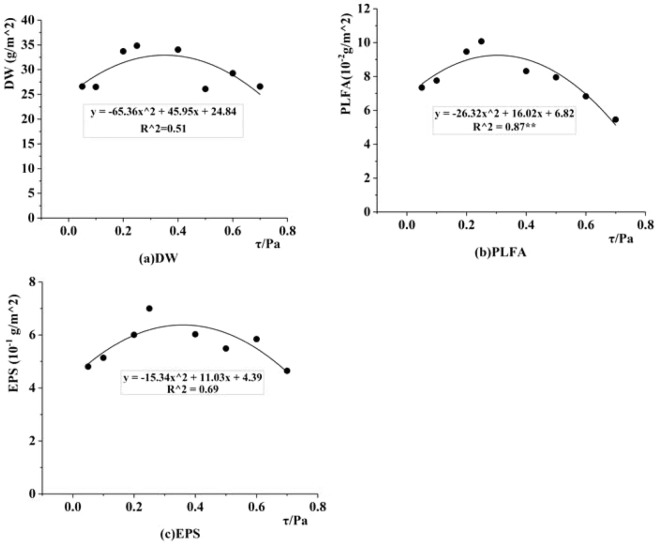
Correlation Diagram between Shear Force and Biofilm Composition in Dynamically Stable Phase.

However, the formation process of biofilms is the result of a combined action of microorganisms, extracellular secretions, particulate matter, nutrients and other substances. The growth of biofilm has a good quadratic correlation with shear force and shows a concave trend with shear force (Fig. 8). Therefore, this paper finds that the optimal shear force range for biofilm growth is [0.2, 0.35] Pa, which indicates that biofilm growth can be effectively inhibited when the shear force is higher or below this shear force range. However, the reasons that shear forces above this range and below this range can inhibit the biofilm growth are different. When there is a relatively large shear force, the migration of the substance is faster and the renewal of microorganisms is also faster, which makes it easy to form a large surface biofilm with relatively high possibility of random^9^. Due to the of the surface biofilm, it is easy to form a biofilm with a small thickness under a relatively large shear force. When the shear force is relatively small, the migration speed of substance is slow, not only reducing the probability of collision and adhesion between microorganisms and particles in biofilm, but also causing an insufficient supply of nutrients required for microbial growth^31,34^, which further reduces the amount of biofilm adhering to and growing on the surface of the medium. At the same time, the ability of microorganisms in biofilms to secrete polysaccharides is poor, reducing the adhesion between biofilm particles and resulting in loose, unstable and an easily detached biofilm structure. In summary, this paper fully expounds the mechanism of how water shear forces influence the attached biofilm. At the same time, there is sufficient evidence to prove that there is a control threshold for the influence of water shear force on biofilm growth process: [0.2, 0.35] Pa, and that biofilm growth can be effectively inhibited when the shear force is higher or below this shear force range.

**Figure 9:**
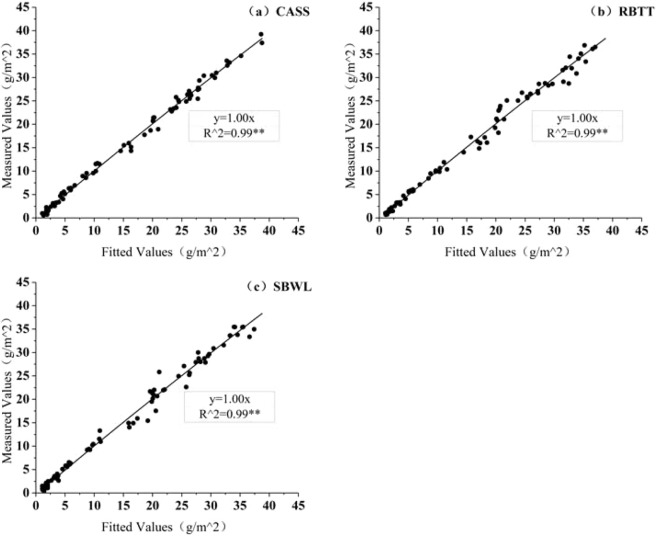
Correlation Diagram between Measured and Fitted Values of Dry Weight of Biofilm.

In addition, this paper uses the Logistic growth model as a prototype to establish a kinetic model for the growth of biofilms. As shown in Fig. 9, the model has higher precision, and the growth process of DW, EPS and PLFAs of biofilm shows a trend of “S-type” growth and can be divided into three phases: adaptive phase, rapid growth phase and dynamically stable phase. This is highly consistent with the findings of Zhou^30^. Winpenny^36^ has from the perspective of microorganisms studied the conditions and time series of surface biofilm formation in biological water supply systems: adhesion, growth, detachment, adhesion again. This cycle is performed repeatedly to form a stable community. This proves that most of the growth processes of attached biofilms on wall of pipeline show a similar trend: microorganisms, solid particles, organic matter and other substances in reclaimed water begin to adhere on the wall of the runner through the viscous extracellular polymeric substance secreted by microorganisms and continuously adsorb or capture solid particles and microbial communities to form an attached biofilm structure getting stable gradually. First, the amount of microbes as well as extracellular polymers secreted by microbes in the attached biofilm is small, and the adhesion capacity of the attached biofilm is not strong. After that, the number and types of microorganisms in biofilm increase rapidly, and viscous secretion increases, adsorbing or capturing solid particles and microbial groups to form biofilms, which is accompanied with detachment of biofilms at the same time. However, the net growth of biofilms during this phase is greater than the amount of, leading to the overall performance of rapidly increasing solid particles and components in biofilm. Finally, after the biofilm reaches the limit thickness, the nutrients needed by microbes to keep growing increase and the microbes begin to compete. As the biofilm thickness increases, the transmission of nutrients inside the biofilm becomes more difficult, resulting in a reduced concentration of nutrients in the biofilm. This causes the decrease or death of internal microbial metabolism. Some attached biofilms will also fall off under the action of external forces such as water shearing force. The quantity of microorganisms gradually tend to be the maximum capacity of the environment, and the biofilms tend to be dynamically stable.

## Conclusion

From this paper, the following main conclusions could be drawn:

(1) Using reclaimed water, the growth process of DW, EPS and PLFAs of the attached biofilm on the inner wall of the drip irrigation system shows a trend of “S-type” growth which could be divided into three phases: adaptive phase, rapid growth phase and a dynamically stable phase.

(2) The influence of water shear forces on biofilm is dual. When there is a relatively large shear force, biofilms with large surface are likely to fall off randomly, which makes it easy to form biofilms with small thickness. When the shear force is relatively small, the migration speed of microorganisms and nutrients are limited, and the ability of microorganisms to secrete polysaccharides is reduced, so that the nutrients needed for microbial growth are insufficient and the adhesion between particles is also reduced. This results in loose, unstable and an easily detached biofilm structure.

(3) By taking the Logistic growth model as the prototype and comprehensively considering the influence of water shear forces on the biofilm, this paper has established a growth model which can describe the growth kinetics of the attached biofilm components (DW, PLFAs and EPS) and can provide reference information for monitoring the occurrence time of biofilm formation processes.

## Supplementary information

Supplementary Information.

## References

[1] Liu HJ (2009). Effect of drip irrigation with reclaimed water on emitter clogging. Transactions of the Chinese Society of Agricultural Engineering.

[2] Brenes MJ, Hills DJ (2001). Microirrigation of wastewater effluent using drip tape. Applied Engineering in Agriculture..

[3] Yan DZ (2009). Biofilm structure and its influence on clogging in drip irrigation emitters distributing reclaimed wastewater. Journal of Environmental Science..

[4] Li YK (2012). Surface topographic characteristics of suspended particulates in reclaimed wastewater and effects on clogging in labyrinth drip irrigation emitters. Irrigation Science..

[5] Zhou B (2013). Quantitative relationship between biofilms components and emitter clogging under reclaimed water drip irrigation. Irrigation Science..

[6] Ravina EP (1992). Control of emitter clogging in drip irrigation with reclaimed wastewater. Irrigation Science..

[7] Song P (2017). Chlorination with lateral flushing controling drip irrigation emitter clogging using reclaimed water. Transactions of the Chinese Society of Agricultural Engineering..

[8] Markku JL (2006). The effects of changing water flow velocity on the formation of biofilms and water quality in pilot distribution system consisting of copper or polyethylene pipes. Water Research..

[9] Saur T (2017). Impact of wall shear stress on initial bacterial adhesion in rotating annular reactor. Plos One..

[10] Characklis, W. G., Wilderer P.A. Structure and Function of Biofilms. Wiley, New York, 6–9 (1989).

[11] Rittmann BE (1982). The effect of shear stress on biofilm loss rate. Biotechnology and Bioengineering..

[12] Characklis, G. & Marshall, K. C. Biofilms. Wiley, New York. (1990).

[13] Stoodley P, Lewandowski Z, Boyle D, Lappin-Scott HM (1999). Structural deformation of bacterial biofilms caused by short-term fluctuations in fluid shear: an *in situ* investigation of biofilm rheology. Biotechnology and Bioengineering..

[14] Liu Y, Tay JH (2002). The essential role of hydrodynamic shear force in the formation of biofilm and granular sludge. Water Research..

[15] Rittmann B. E. & McCarty P. L. Environmental Biotechnology: Principles and Applications. Mc-Graw Hill, New York (2001).

[16] Mathieu L (2014). Drinking water biofilm cohesiveness changes under chlorination or hydrodynamic stress. Water Res..

[17] Van Loosdrecht MCM (1995). Biofilm structures. Water Science and Technology..

[18] Ochoa JC (2007). Influence of non-uniform distribution of shear stress on aerobic biofilms. Chemical Engineering Science..

[19] Bakke R (1984). Activity of pseudomonas aeruginosa in steady-state biofilms. Biotechnology and Bioengineering..

[20] Chang HT (1991). Biofilm detachment mechanisms in a liquid fluidized bed. Biotechnol Bioeng..

[21] Nicolella C, Felice RD, Rovatti M (2000). An experimental model of biofilm detachment in liquid fluidized bed biological reactors. Biotechnol Bioeng..

[22] Christensen B. E. & Characklis W. G. Physical and chemical properties of biofilms. In: Characklis, WG, Marshall, KC, editors, Biofilms. New York (NY): John Wiley; 93–130 (1990).

[23] Paul E, Ochoa JC, Pechaud Y (2012). Effect of shear stress and growth conditions on detachment and physical properties of biofilms. Water Res..

[24] Li GB (2011). Effects of average velocity on the growth and surface topography of biofilms attached on the reclaimed wastewater drip irrigation system laterals. Irrigation Science..

[25] Percival SL (1998). Biofilms, mains water and stainless steel. Water Research..

[26] Peter JO, Peter MH, Robin MS (2003). Factors affecting biofilm accumulation in model distribution systems. Journal American Water Works Association..

[27] Zhu K. Q. & Xu C. X. Viscous Fluid Mechanics. Beijing: Higher Education Press (2009).

[28] Chen Y.H. The Design of Intelligent Drip Irrigation Network Control System. International Conference on Internet Technology & Applications. IEEE. (2011).

[29] Richards FJ (1959). A flexible growth for expirical use. J. Exp. Botany..

[30] Bo Z (2016). A kinetic model for biofilm growth inside non-PC emitters under reclaimed water drip irrigation. Agricultural Water Management..

[31] Park A (2011). Effect of shear stress on the formation of bacterial biofilm in a microfluidic channel. BioChip J..

[32] Guo Y (2013). Fabrication and Characteration of Superhydrophobic Polymer/hydrophobic SiO_2_ Composite Coatings. Chinese. Journal of Materials Research..

[33] Wilen BM, Jin B, Lant P (2003). The influence of key chemical constituents in activated sludge on surface and flocculating properties. Water Research..

[34] Ramasamy P, Zhang XT (2005). Effects of shear stress on the secretion of extracellular polymeric substances in biofilms. Water Science &. Technology..

[35] Tay JH, Liu QS, Liu Y (2001). The effects of shear force on the formation, structure and metabolism of aerobic granules. Appl Microbiol Biotechnol.

[36] Winpenny J (1996). Ecological Determinants of Bioflim Formation. Biofouling..

