# Supplementary material for: Accumulation mechanism of biofilm under different water shear forces along the networked pipelines in a drip irrigation system
Source: Sci Rep. 2020 Apr 24;10:6960. doi: 10.1038/s41598-020-63898-5 (PMC7181636; doi:10.1038/s41598-020-63898-5)
Supplement: Supplementary file 1 — Supplementary Information. [file 41598_2020_63898_MOESM1_ESM.doc]

**Supporting Information**

**Accumulation mechanism of biofilm under different water shear forces along the networked pipelines in a drip irrigation system**

Zucheng Guo^1^, Tianzhi Wang^2,3, *^, Yaojie Shen^1^, Zhimei Cui^1^, Alex Goodwin^4^

*Corresponding author: Tianzhi Wang

1. China Everbright International LTD, Hongkong, 999077, China

2. Environmental Simulation and Pollution Control State Key Joint Laboratory, School of Environment, Tsinghua University, Beijing 100084, PR China

3. College of Water Resources and Civil Engineering, China Agricultural University, Beijing 100083, China

4. Department of Agricultural and Biological Engineering, University of Illinois at Urbana-Champaign, Urbana, IL 61801, USA

* Corresponding author: Tel.: +8617695588053, E-mail address: wangtianzhi@tsinghua.edu.cn

Zucheng Guo, Tianzhi Wang and Yaojie Shen were equally contributed to this paper.

**Background and introduction of biofilm cultivation system designed by our group:**

(1) In conventional experiment, destructive sampling was usually taken in order to obtain clogging substance from the inner of networked pipelines in drip irrigation system. However, the destructive sampling would reduce the real amount of clogging substance and might make experimental error because it is very difficult to peel off the networked pipelines and obtain the clogging substance.

(2) It is almost impossible to see the inner flow path intuitively and control hydrodynamic conditions in emitters during drip irrigation system running. So it is very difficult to make clear the effects of a certain factor (especially flow velocity and wall shear force) on clogging substance if using conventional drip irrigation system. However, the hydrodynamic conditions have a direct impact on formation of clogging substance at the beginning.

(3) In order to achieve to control hydrodynamic condition in networked pipelines and eliminate the above experimental error, our group designed a clogging substance cultivation system. And the system had accessed to National Invention Patent in 2014:

Li Yunkai, Tianzhi Wang, Feng Ji, Pei Yiting. Simulator and its usage of biofilm attached on the wall surface of drip irrigation system. [P]. China: ZL2012104552806, 2014

(4) There are some advantages of the simulator. First, the hydrodynamic conditions could be controlled in this simulator. The simulator uses a unique design of inner-outer cylinder combining with a variable speed motor. Hydrodynamic shear force would be occurred on slice’s surface as the inner cylinder rotates to drive the water flow. The τ could be calculated accurately by Equation 4 in this experiment.

$\omega_{1}=\frac{\tau\times1200}{6080.25}\times10^{3}$ （4）

Secondly, the sampling rack is designed by modular concave bed in sample groove (Part 19, showed in fig.2), which could increase the stability and sampling speed in the process of cultivation. Samples would be protected and the integrity of samples could also be improved significantly as the sampling rack (Part 9, showed in fig.6) could be detachable from the simulator. Thirdly, the growth process of clogging substance could be seen intuitively.


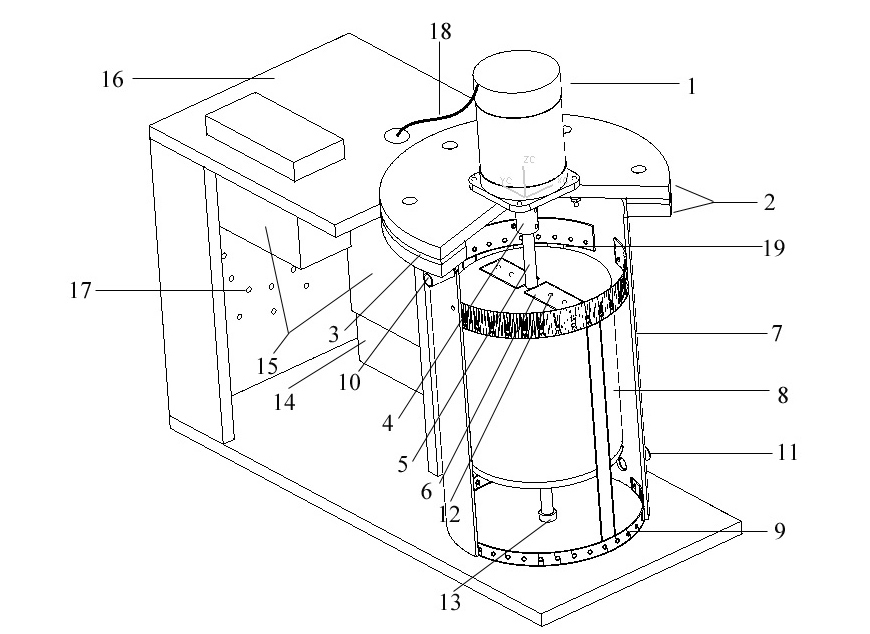


Note: 1-motor, 2-flanges, 3-gasket, 4-connect bearing, 5-motor shaft, 6- fixing steel sheet, 7-outer cylinder, 8-inner cylinder, 9-sampling rack, 10-water outlet, 11-water inlet, 12-fixing screws, 13-fixing bearing, 14-transformer, 15-rotating speed controller, 16-power distribution box, 17-vents, 18-wire, 19-sample groove.

Fig.S1 Overall drawing of cultivation simulator


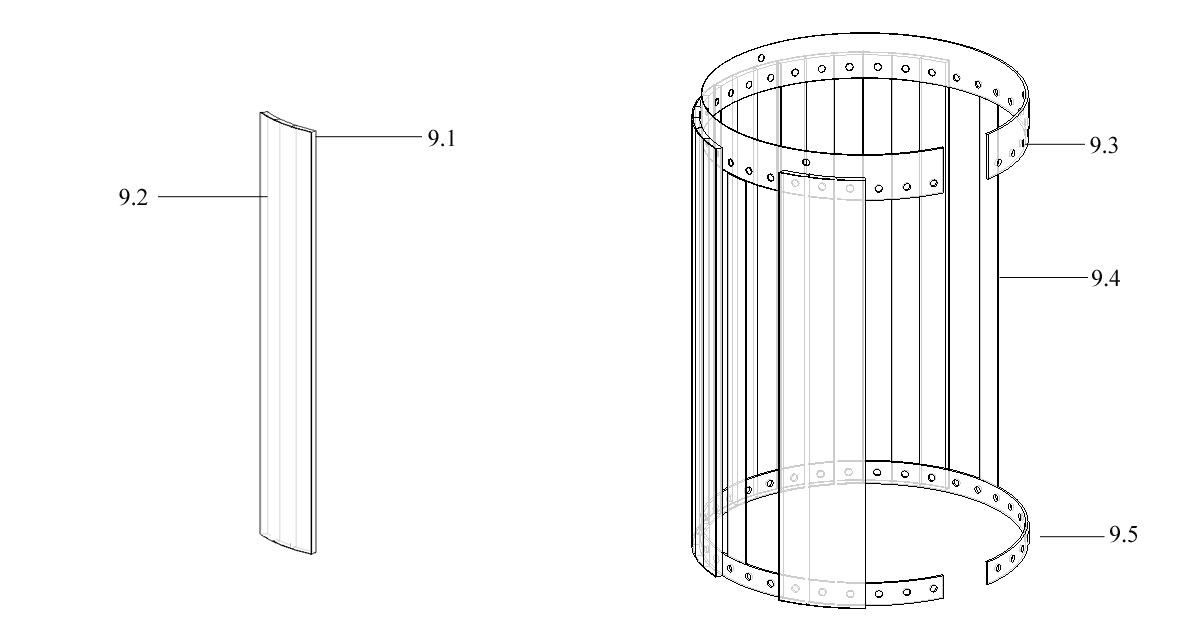


Note: 9.1-signal PE slice, 9.2-sample unit, 9.3-screw hole, 9.4-steel support, 9.5-steel ring.

Fig.S2 Overall drawing of sampling rack (Part 9)


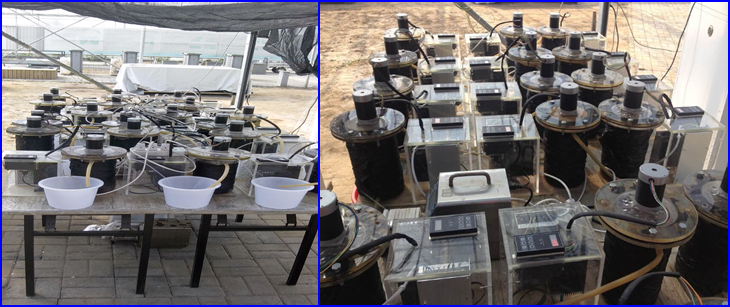


Fig.S3 Overall drawing and images of clogging substance cultivation system systems (photos complements)
